# Chikungunya E2 Protein Produced in *E. coli* and HEK293-T Cells—Comparison of Their Performances in ELISA

**DOI:** 10.3390/v12090939

**Published:** 2020-08-26

**Authors:** Flávia Fonseca Bagno, Lara Carvalho Godói, Maria Marta Figueiredo, Sarah Aparecida Rodrigues Sérgio, Thaís de Fátima Silva Moraes, Natália de Castro Salazar, Young Chan Kim, Arturo Reyes-Sandoval, Flávio Guimarães da Fonseca

**Affiliations:** 1 Centro de Tecnologia em Vacinas (CT-Vacinas), Parque Tecnológico da UFMG (BH-Tec), Universidade Federal de Minas Gerais (UFMG), Belo Horizonte-MG 31320-000, Brazil; flavia.bagno@gmail.com (F.F.B.); lcarvalhogodoi@gmail.com (L.C.G.); mariamartafigueiredo@gmail.com (M.M.F.); sarsergio36@gmail.com (S.A.R.S.); thais.moraes00@hotmail.com (T.d.F.S.M.); natsalazar@gmail.com (N.d.C.S.); 2 Laboratório de Virologia Molecular e Aplicada, Departamento de Microbiologia, Instituto de Ciências Biológicas (ICB/UFMG), Belo Horizonte-MG 31270-901, Brazil; 3 Colégio Técnico da Universidade Federal de Minas Gerais (COLTEC), Belo Horizonte-MG 31270-901, Brazil; 4 The Jenner Institute, Nuffield Department of Medicine, The Henry Wellcome Building for Molecular Physiology, Roosevelt Drive, University of Oxford, Oxford OX3 7DQ, UK; young.kim@some.ox.ac.uk (Y.C.K.); arturo.reyes@ndm.ox.ac.uk (A.R.-S.)

**Keywords:** chikungunya virus, envelope protein 2, ELISA, heterologous expression, *E. coli*, HEK293-T cells

## Abstract

Chikungunya virus (CHIKV) is a mosquito-borne pathogen that causes a disease characterized by the acute onset of fever accompanied by arthralgia and intense joint pain. Clinical similarities and cocirculation of this and other arboviruses in many tropical countries highlight the necessity for efficient and accessible diagnostic tools. CHIKV envelope proteins are highly conserved among alphaviruses and, particularly, the envelope 2 glycoprotein (CHIKV-E2) appears to be immunodominant and has a considerable serodiagnosis potential. Here, we investigate how glycosylation of CHIKV-E2 affects antigen/antibody interaction and how this affects the performance of CHIKV-E2-based Indirect ELISA tests. We compare two CHIKV-E2 recombinant antigens produced in different expression systems: prokaryotic-versus eukaryotic-made recombinant proteins. CHIKV-E2 antigens are expressed either in *E. coli* BL21(DE3)—a prokaryotic system unable to produce post-translational modifications—or in HEK-293T mammalian cells—a eukaryotic system able to add post-translational modifications, including glycosylation sites. Both prokaryotic and eukaryotic recombinant CHIKV-E2 react strongly to anti-CHIKV IgG antibodies, showing accuracy levels that are higher than 90%. However, the glycan-added viral antigen presents better sensitivity and specificity (85 and 98%) than the non-glycosylated antigen (81 and 71%, respectively) in anti-CHIKV IgM ELISA assays.

## 1. Introduction

Chikungunya virus (CHIKV, Family Togaviridae) is a re-emerging alphavirus that causes clinical manifestations characterized by febrile illness associated with arthralgia and skin rash. Additionally, it has been associated with cases of meningoencephalitis, Guillain–Barré syndrome, and hemorrhagic disease [1]. The combination of IgM and IgG ELISAs may provide evidence for both recent and past exposures to the virus [2]. Several commercial tests are available for anti-CHIKV antibody detection. Some of them were evaluated by reference laboratories, and three recently tested IgM ELISA kits showed acceptable performances [3]. An IgM capture Enzyme-Linked Immunosorbent Assay (MAC-ELISA), developed by the US Centers for Disease Control and Prevention (CDC), uses whole virus antigens [4]; however, complete CHIKV particle inactivation with no loss of antigenic properties requires gamma-irradiation and lyophilization for long-term storage [5]. Consequently, the cost of antigen production from CHIKV cultures can be high, and many of the commercially available ELISA kits are considered expensive indeed [6].

Generally, protein-based ELISAs for the detection of viruses are based on either surface glycoproteins or capsid proteins. Surface glycoproteins tend to be highly antigenic and major inducers of neutralizing antibodies but, because such proteins have the addition of sugar moieties, the biochemically complete recombinant protein only can be produced in eukaryotic systems [7]. Considering the structural antigens of CHIKV, the envelope proteins E1 and E2 are the ones most frequently included in serological diagnostic kits [8]. The E2 protein appears to be immunodominant and has a higher serodiagnostic potential when compared to E1 [8,9,10]. Studies with other alphaviruses have shown that neutralizing antibodies are generally directed against E2 and, to a lesser extent, to E1 during infection [11,12].

Currently, there are many different platforms for recombinant antigen production, and they range from bacteria [13] and yeasts [14,15] to higher eukaryotic cells in culture [16,17]. The choice of a recombinant platform for protein production has to take many aspects into consideration, including cost-effectiveness. The expression of CHIKV envelope proteins in *E. coli* or in eukaryotic systems and their diagnostic potential have been described [8,18,19,20,21,22], however, no previous study has directly compared the performance of such antigens produced in two different expression systems, by ELISA.

*Eescherichia coli*-based expression systems are frequently the first option for recombinant protein production due to its low cost, well-known biochemistry and genetics, rapid growth, and good productivity. Nonetheless, disadvantages of this system include the lack of post-translational modifications (such as glycosylation, disulfide phosphorylation, or proteolytic processing), inclusion body formation, and endotoxin production. Conversely, mammalian cells have the ability to express complex recombinant proteins with proper folding and glycosylation. A drawback, however, is the introduction of the exogenous gene to cells may be time-consuming and more complex, increasing the overall cost of protein production in comparison to prokaryotic expression systems [23].

During CHIKV replication, E2 is post-translationally N-glycosylated at amino acid residues 263 and 345 [24]. We know from past studies that glycosylation can influence the function, structure, antigenicity, and immunogenicity of various viral glycoproteins [25,26,27]. Considering that E2 is likely necessary for a diagnostic platform to detect antibodies against CHIKV, we ask how production of recombinant E2 in either prokaryotic or eukaryotic expression platforms would impact the performance of such antigens in enzyme-linked immunosorbent assays (ELISA). To look into that, we produce recombinant CHIKV-E2 proteins in either prokaryotic or eukaryotic systems and compare their use in Indirect ELISA tests.

## 2. Materials and Methods

### 2.1. Design and Production of CHIKV-E2 Protein in E. coli

Coding sequences for CHIKV-E2 were collected from the NCBI genes database, aligned using MEGA7 software, and analyzed to create a consensus sequence. The transmembrane domain was removed, and the gene was codon-optimized for expression in *E. coli*. The nucleotide sequence of the truncated protein was commercially synthesized and subcloned into the pET-21 expression vector, which included a histidine tag to the construction.

The pET-21 vector containing the gene of interest was used to transform *E. coli* BL21(DE3) strain by heat shock. Plasmid-positive clones were induced with IPTG (0.5 mM) at three different conditions (18 °C, overnight/25 °C, overnight/37 °C, 4 h), and the cells were harvested by centrifugation and resuspended in an appropriate volume of lysis buffer (20 mM Tris-HCl, 500 mM NaCl, 1 mM PMSF, 5 mM Benzamidine and 5 mM DTT). Cells were disrupted using a high-pressure homogenizer Emulsiflex and the lysate was separated by centrifugation into soluble fractions (supernatant) and insoluble fractions (pellet). The pellet was resuspended in buffers with different urea concentrations (2, 4, 6 and 8 M) to evaluate the solubility of the recombinant protein and the fractions were examined by SDS-PAGE. The antigen was purified by affinity chromatography using nickel columns in an ÄKTA prime plus system (GE Healthcare, USA) and eluted with 500 mM of imidazole.

### 2.2. Design and Production of CHIKV-E2 Protein in HEK-293T

The production of recombinant CHIKV-E2 protein in the eukaryotic system was carried out as described previously [28]. Briefly, the codon-optimized gene of CHIKV-E2 (a.a. 1–346) was cloned into the pHLsec vector, which is flanked by the chicken β-actin/rabbit β-globin hybrid promoter with a signal secretion sequence and a Lys-His6 tag.

The pHLsec CHIKV-E2 plasmid (500 µg) was transfected into HEK-293T cells using polyethyleneimine (PEI) in roller bottles under standard cell culture conditions. Five days after, transfection cells were discarded, and the media was filtered through 0.22 µM disposable filters. The secreted protein was purified from the supernatant by affinity chromatography using nickel columns (HisTRAP^TM^, GE Healthcare, Chicago, IL, USA), in an ÄKTA chromatography system and eluted with Imidazole 500 mM. Glycosylation on the recombinant protein was assessed by incubating the purified antigen with the PNGase F enzyme, which removes all N-linked glycans, followed by western blot analysis using anti-CHIKV mouse serum.

### 2.3. Sera Bank and Ethical Considerations

All human sera used in this work were previously tested by the Central Laboratory of Public Health (*Laboratório Central de Saúde Pública*—LACEN) at the Ezequiel Dias Foundation (*Fundação Ezequiel Dias*—FUNED), in Belo Horizonte, MG, Brazil, according to directives from the Brazilian Ministry of Health (BMH). Samples included CHIKV-positive sera and CHIKV-negative sera and were collected from healthy donors or from CHIKV-infected patients. Infected patients had clinical symptoms compatible to Chikungunya Fever and underwent laboratory diagnosis using an anti-Chikungunya IgG ELISA kit (Euroimmun, Germany, ref. EI293aG). [29]. Written consents for individual serum were not obtained because all serum samples belonged to the LACEN-FUNED bio-repository. The use of samples was approved by the LACEN-FUNED’s research board.

### 2.4. ELISA

Detection of anti-E2 IgG antibodies in sera was done using the Indirect ELISA method (enzyme-linked immunosorbent assay) using CHIKV-E2 produced in either bacteria or HEK193 cells as antigens. High binding plates of polystyrene (Corning-Costar, Corning, NY, USA) were coated overnight at 4 °C with the antigens diluted in a carbonate buffer (pH 9.6). The wells were blocked with bovine serum albumin (BSA) for 2 h at 25 °C. Regarding each assay, 100 μL of serum diluted in Phosphate Buffered Saline with Tween 20 (PBS-T) were added and incubated for 45 min for IgG detection or 60 min for IgM detection at 37 °C. After five washes with PBS-T, 100 μL of conjugated [Anti-human IgM (A0420) or Anti-human IgG (A0170), Sigma] diluted in PBS-T were added. Plates were incubated 30 min for IgG detection or 45 min for IgM detection at 37 °C, washed five times and 100 μL of TMB (3,32′,5,5;-tetramethylbenzidine—Moss, USA) were added and incubated for 15 min (test revealing). Last, 100 μL of 0,5 M H_2_SO_4_ solution was added to the wells to stop the reaction. Plates were analyzed in a Microplate Reader at an optical density (O.D.) of 450 nm.

The optimal concentrations of the well-coating recombinant proteins were determined based on a clear distinction of anti-CHIKV antibodies using positive and negative samples (ratio).

Cut-off values were based on the Receiver Operating-Characteristics (ROC) curve analysis.

An index (*I*) of sample absorbance (Abs) over the value of cut-off was calculated, according to the Equation (1):

(1) $$I=\;\frac{Abs_{450nm}\;}{cut-off\;value}.$$

Results were classified as follows:

*I* < 0.9: negative

0.9 ≤ *I* < 1.1: borderline

*I* ≥ 1.1: positive

A total of 158 samples previously characterized, including CHIKV-positive sera (*n* = 70) and CHIKV negative sera (*n* = 88) were tested against both antigens. Statistical analyses were performed using GraphPad Prism (version 8.0.2) and MedCalc (https://www.medcalc.org/). Sensitivity, specificity, accuracy, area under the ROC curve, and positive and negative predictive values were calculated and compared to the reference kit [anti-Chikungunya IgG ELISA kit (Euroimmun, Germany, ref. EI293aG)] to evaluate the performance of either antigen [30]. Borderline results were not included. Agreements between the tests were assessed by calculating Cohen’s Kappa coefficient (k) and interpreted as follows: values ≤ 0 indicating no agreement; 0.01–0.20 had none to slight; 0.21–0.40 had fair; 0.41–0.60 had moderate; 0.61–0.80 had substantial; and 0.81–1.00 had almost perfect agreement [31]. Chi-squared tests were performed to compare proportions of sensitivity, specificity, and accuracy for both antigens [30,32].

## 3. Results

### 3.1. Production of CHIKV-E2 Protein in E. coli

**Figure 1:**
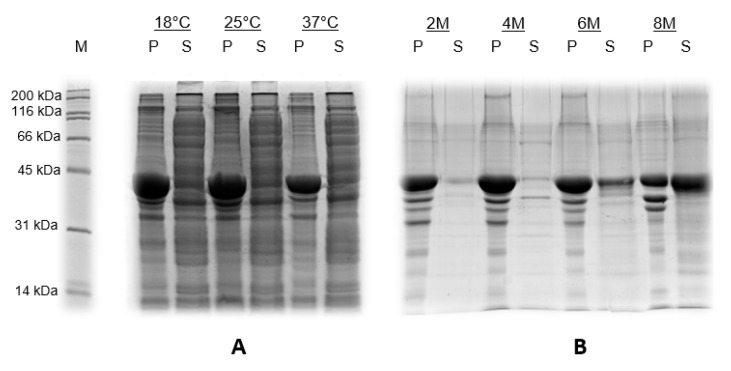
Expression and solubility of the recombinant CHIKV-E2 protein produced in *E. coli* BL21 (DE3). (**A**) Recombinant CHIKV-E2 produced in the pellet (P, insoluble) and supernatant (S, soluble) fractions at different induction temperatures (18, 25 and 37 °C). (**B**) Solubility of the recombinant protein at different concentrations of urea (2, 4, 6 and 8 M).

After the induction at different temperatures (18, 25 and 37 °C), we analyzed the pellet (P, insoluble) and supernatant (S, soluble) fractions by SDS-PAGE (Figure 1A). We also evaluated the solubility of the recombinant protein at different concentrations of urea (2, 4, 6 and 8 M) (Figure 1B).

Concerning all three temperatures, the prokaryotic CHIKV-E2 protein (42 kDa) was expressed as inclusion bodies, remaining in the pellet fractions (Figure 1A). Regarding the urea concentration needed to solubilize the recombinant proteins, it was necessary to resuspend the pellet in an 8 M urea buffer, indicating that the product was highly insoluble (Figure 1B).

### 3.2. Purification of the Recombinant Proteins

**Figure 2:**
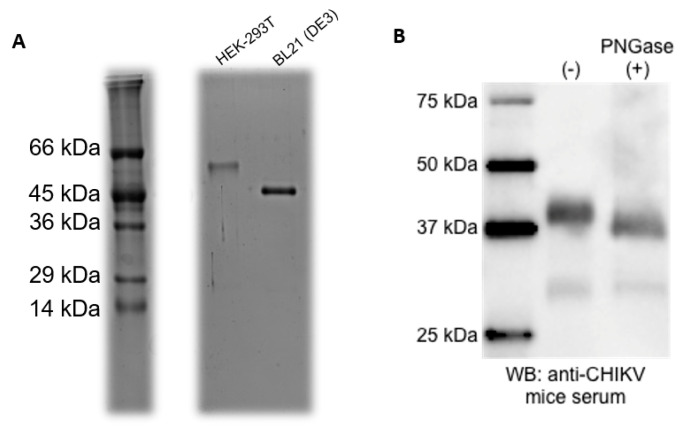
Comparison of molecular mass and glycosylation analysis in CHIKV-E2 from HEK-293T. (**A**): Supernatant from HEK-293T cells and pellets from BL21 (DE3) cells were purified by affinity chromatography and evaluated in SDS-PAGE. (**B**): CHIKV-E2 produced in HEK-293T cells was treated with a PNGase F to remove all N-linked glycans and analyzed on western blot using anti-CHIKV mouse serum, showing that the higher molecular mass from the eukaryotic antigen is a result of protein glycosylation.

CHIKV-E2 proteins produced in HEK-293T cells were purified straight from the culture supernatant, whereas the antigens produced in BL21 (DE3) *E. coli* were recovered from inclusion bodies after cell lysis. Both fractions were submitted to affinity chromatography using Ni columns in the ÄKTA system. SDS-PAGE from purified aliquots showed unique bands of the expected molecular mass (Figure 2A). A higher molecular mass was observed for CHIKV E2 protein produced in HEK-293T cells and this could be due to the protein glycosylation. To confirm this, CHIKV-E2 produced in HEK-293T cells was treated with PNGase F to remove N-linked glycans, followed by western blot using anti-CHIKV mouse serum. PNGase F-treated CHIKV-E2 antigen showed a decrease in molecular mass, suggesting that the higher molecular mass in the eukaryotic antigen was indeed a result of protein glycosylation (Figure 2B).

### 3.3. Standardization of ELISA Using Recombinant CHIKV-E2 from Prokaryotic and Eukaryotic Systems

**Figure 3:**
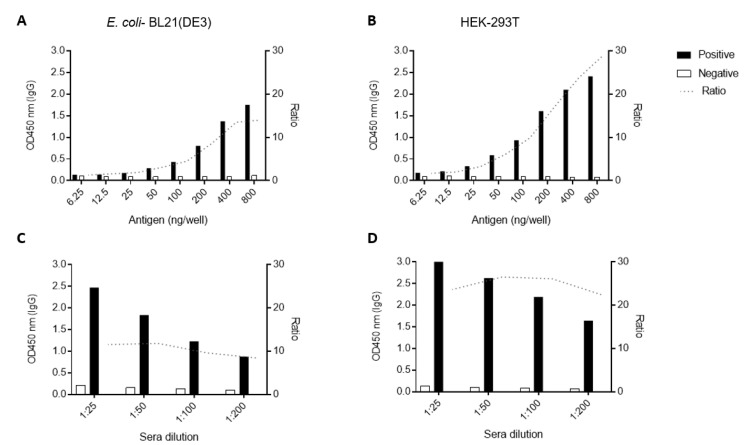
Standardization of the Indirect IgG ELISA to detect antibodies against CHIKV. Different amounts (titer) of antigens (**A**,**B**) and different pooled sera dilutions (**C**,**D**) were tested in the Indirect ELISAs to determine minimum optimal antigen concentrations and maximum optimal sera dilution. Graphics at the left show the results for CHIKV-E2 produced in *E. coli* and at the right show results using the eukaryotic recombinant antigen.

Initially, we tested different amounts of antigen per well using CHIKV-positive and -negative pools of sera (ten samples in each pool). Considering anti-CHIKV IgG detection, parameters were the same for both antigens. We determined the use of 200 ng of antigen/well as the minimal optimal antigen titer based on the satisfactory distinction (Ratio > 4) between the positive and negative samples’ absorbances (Figure 3A,B). Likewise, we established the 1:100 serum sample dilution as the optimal dilution to discriminate negative and positive sera pools as it showed a 26 and ten-fold ratio for HEK-293T- and *E. coli*-made E2 (Figure 3C,D), respectively.

**Figure 4:**
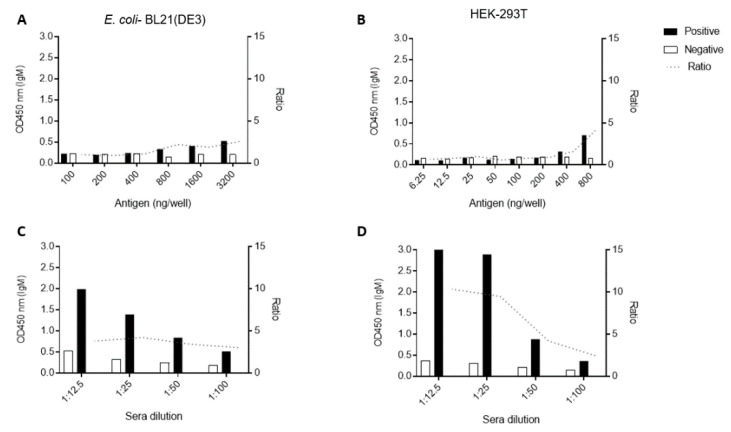
Standardization of the Indirect IgM ELISA to detect antibodies against CHIKV. Different amounts (titer) of antigens (**A**,**B**) and different pooled sera dilutions (**C**,**D**) were tested in the Indirect ELISAs to determine minimum optimal antigen concentrations and maximum optimal sera dilution. Graphics at the left show the results for CHIKV-E2 produced in *E. coli* and at the right show results using the eukaryotic recombinant antigen.

Regarding the anti-IgM ELISA, we first titrated both antigens using the 1:100 sera dilution and different ranges of antigen concentrations (Figure 4A,B). Concerning the case of the bacteria-made protein, we began by using some lower concentrations as we tested for the anti-IgG ELISA (Figure 3A), but the initial small concentrations of 6.25–50 ng of protein/well rendered no separation between positive and negative sera samples. When we varied sera dilutions, best positive-to-negative separation ratios were obtained using a 1:25 dilution. HEK-293T antigens presented a higher sensitivity than the *E.coli* antigen for the IgM assays, as less antigen was needed to get a ratio ≥ 4. Therefore, we determined that 3200 ng/well of E2 produced in *E. coli* (Ratio = 4, Figure 4C) and 200 ng/well of E2 from HEK-293T cells (Ratio = 9, Figure 4D) were the best amounts of protein to be used in the respective ELISAs.

### 3.4. Performance of CHIKV-E2 from Prokaryotic and Eukaryotic Systems as Antigens in ELISA

**Figure 5:**
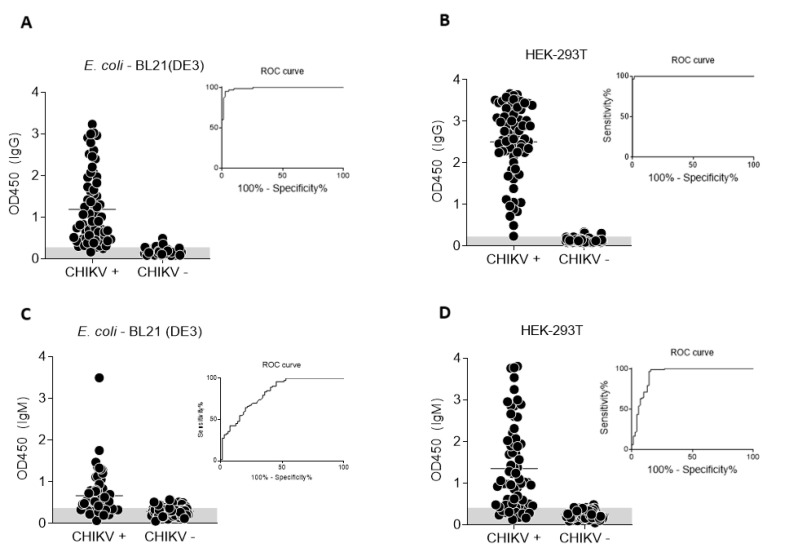
Performance comparison of Indirect ELISAs using either CHIKV-E2 antigen produced in *E. coli* or HEK-293T cells. Individual CHIKV-negative or -positive sera samples from a characterized sera bank were used to evaluate Indirect ELISAs employing antigens produced in prokaryotic (**A**,**C**) or eukaryotic cells (**B**,**D**). Each dot represents an individual serum sample. The ROC curve and observed specificity for the tests are shown as insets on each panel.

We analyzed the performance of both antigens in recognizing anti-CHIKV antibodies from individual samples and compared results to those obtained with a commercially available serological test. The ELISA results for anti-CHIKV IgG and IgM detection in individual sera from our sera bank (dot plot), as well as the ROC curve for each assay, are shown in Figure 5.

The results were compared to the reference ELISA kit (Euroimmun). A summary of the results for each assay, including cut-off values, sensitivity, specificity, positive predicted value (PPV) negative predicted value (NPV), accuracy (AC) area under the ROC curve (AUC) and Cohen’s kappa index (*k*) with 95% confidence intervals (CI) of each test, is displayed in Table 1.

Regarding IgG detection, the antigens made from *E.coli* or HEK293-T exhibited sensitivities of 97 and 99%, respectively (*p* = 0.402). Both antigens also presented high specificity values: 96% for the *E.coli*-made protein and 97% for the HEK293-T-made antigen (*p* = 0.748). The sensitivity of the test employing the *E.coli*-expressed E2 protein was 81%, whereas the sensitivity of the ELISA using the E2 antigen made in HEK293-T cells was 85% (*p* = 0.564). Regarding the specificity values, the results differed significantly: 71% and 98%, respectively (*p* = 0 < 0.005). Accuracies were 97% for both IgG (*p* = 1.000) assays using the different antigens; however, the accuracy of the IgM test employing the glycosylated protein was 92% against only 76% in the test using the bacteria-expressed E2 (*p* = 0 < 0.005). All results were consistent with the observed AUC values (Table 1), which represent the overall performance of each test. The lower Cohen’s kappa index for the *E.coli*-based IgM-assay (*k* = 0.515) reflects moderate agreement with the reference test, whereas the HEK293-T-based test showed almost perfect agreement (*k* > 0.81).

Then, we compared the results of both antigens in ELISA to evaluate the agreement between them by Cohen’s kappa index (Table 2). The agreement between both antigens is almost perfect (*k* = 0.945) for the IgG assay, however, it was only moderate (*k* = 0.607) when we considered anti-IgM ELISA performances.

## 4. Discussion

Arboviruses are considered as important public health problems, causing large epidemics worldwide. Due to the co-circulation of CHIKV and other arboviruses in tropical regions, there is an urgent need for efficient and reliable diagnostic methods for the detection of these infections. Currently available serological tools to detect Chikungunya infections include IgM and IgG ELISAs, as well as IgM and IgG immunochromatographic tests, and many of those tests are based on recombinant antigens.

Prokaryotic systems are widely used to express recombinant proteins, including CHIKV antigens [18,19,33]. Such platforms have the advantage of being simple, fast and inexpensive. However, most bacterial expression systems are limited in their ability to produce proteins in a soluble manner and may result in the formation of aggregates known as inclusion bodies [34] as we observed for CHIKV-E2 produced in BL21(DE3). To contrast, eukaryotic expression systems, like mammalian cells, usually produce proteins in their native conformation, preserving post-translational modifications, such as glycosylation [35]. Indeed, each expression system has a particular biochemical environment, which affects the production and conformation of proteins. The availability of cofactors, folding machinery and enzymes that introduce post-translational modifications are important for the biological activity of the aimed protein. Therefore, diagnostic tools that include glycan-containing viral antigens (when the original antigen is naturally glycosylated) usually present higher sensitivities and specificities, considering that a substantial proportion of antibodies in infected patients are directed against viral glycan epitopes [36].

We compared the performance of two CHIKV-E2 antigens, one produced in *E. coli* BL21(DE3) cells (non-glycosylated) and other produced in HEK-293T mammalian cells (glycosylated). Regarding anti-CHIKV IgG detection, we have used the same protein concentrations per well and the same serum dilution in tests using both recombinant antigens and observed comparable performances. These results suggest that, for an Indirect anti-CHIKV IgG ELISA, the antigen produced in prokaryotic cells have a similar performance to the protein expressed in mammalian cells (Figure 5A,B and Table 2). Therefore, we consider that the *E. coli*–made CHIKV-E2 represents the best cost-benefit option (regarding a cost-performance ratio). Conversely, the glycosylated protein produced in HEK-293T cells presented a significant improvement to detect anti-CHIKV IgM antibodies when compared to the antigen produced in *E. coli* (*p* = 0 < 0.005 comparing the accuracies, 92% and 76%, respectively). Concerning the anti-IgM assay, we opted to use less diluted sera samples (1:25) as better CHIKV positive-to-negative sera differentiation was obtained when 200 ng/well of HEK-293T-expressed antigens were used (the same amount determined to be the best option in the anti-IgG ELISA). Nonetheless, even when using smaller sera dilution, we only achieved effective sera differentiation when 3200 ng of bacteria-made antigen per well was used. This indicates that the eukaryotic-made antigen is far superior to the *E.coli* antigen for IgM assays since less antigen is needed. Nonetheless, a better ELISA prototyping remains to be conducted, as most commercially available anti-CHIK ELISAs employ the 1:100 sera dilution for the assays [37].

Taking the results together, one question stands out: why does the difference in accuracy between the two antigens become more obvious only in the IgM assay? This can be speculated in two ways: first, IgMs have lesser affinities than IgGs and, therefore, are prone to poorly recognize proteins that do not contain molecular characteristics closely resembling the antigens against which they were originated (such as glycan residues) [38]. Second, there is evidence suggesting that IgM class antibodies have a special tendency to recognize glycosylated antigens [39]. To further look into that, however, studies of mutated, non-glycosylated E2 proteins produced in eukaryotic systems would be required.

Glycoproteins are one of the major components of pathogenic viruses [40], and have important roles in infection, immunity, and are fundamental to a wide range of molecular and cellular processes [41]. Recombinant glycoproteins produced in eukaryotic cells are correctly glycosylated and are likely to retain linear and non-linear epitopes that may be readily recognized by a wide range of specific antibodies. The use of such proteins may result in diagnostic systems with higher sensitivities and/or specificities. Nonetheless, these proteins are more complex to produce on an industrial scale and are more expensive upon reaching the market. Conversely, bacteria-made recombinant proteins are much simpler, cheaper, and usually prone to be obtained in higher quantities when compared to eukaryotic-made proteins. However, the lack of post-translational modifications in prokaryotic cells may render proteins that are less efficiently recognized by specific antibodies, particularly when the original protein is glycosylated. This may result in the loss of specificity and/or sensitivity when such proteins are used in diagnostic systems. Therefore, despite the advantages and disadvantages of each expression system, the choice to use eukaryotic- or prokaryotic-made recombinant proteins should be an empirically made decision, considering an equation that includes cost, easiness to produce, and the overall performance of tests employing each protein.

## 5. Conclusions

We analyzed the performance of the CHIKV-E2 antigen produced in *E. coli* compared to the very same protein produced in HEK-293T mammalian cells. Both prokaryotic and eukaryotic recombinant CHIKV-E2 showed a high potential to be recognized by anti-CHIKV IgG antibodies in an Indirect ELISA. Nevertheless, the eukaryotic–made protein presented a much better performance than its bacteria-made counterpart in an Indirect ELISA to detect anti-CHIKV IgM.

## Figures and Tables

**Table 1:** Different parameters to evaluate the diagnostic performance of ELISA tests to detect anti-CHIKV antibodies employing either prokaryotic-made or eukaryotic-made E2 antigens.

		IgG-E.coli			IgG-HEK-293T			IgM-E.coli			IgM-HEK-293T		
**Reference**		Positive	Negative	Sum	Positive	Negative	Sum	Positive	Negative	Sum	Positive	Negative	Sum
	Positive	66	2	68	69	1	70	42	10	52	57	10	67
	Negative	2	55	57	3	83	86	17	42	59	1	76	77
	Sum	68	57	125	72	84	156	59	52	111	58	86	144
**Cut-off**		0.279			0.225			0.328				0.409	
**Sensitivity(95% CI)**		97 (90–100)%			99 (92–100)%			81 (67–90%)			85 (74–93)%		
**Especificity(95% CI)**		96 (88–100)%			97 (90–99)%			71 (58–82)%			98 (93–100)%		
**PPV (95% CI)**		97 (89–99)%			96 (88–100)%			72 (62–79)%			98 (89–100)%		
**NPV (95% CI)**		96 (88–99)%			99 (92–99)%			81 (70–88)%			88 (81–93)%		
**AC 95% CI)**		97 (92–99)%			97 (94–99)%			76 (67–83)%			92 (87–96)%		
**AUC(95% CI)**		0.987 (0.972–1.000)			0.999 (0.998–1.000)			0.817 (0.747–0.888)			0.9251 (0.877–0.974)		
***k*** **(95% CI)**		0.936 (0.873–0.998)			0.948 (0.898–0.998)			0.515 (0.358–0.673)			0.845 (0.758–0.932)		

PPV: positive predicted value, NPV: negative predicted value, AC: accuracy, AUC: area under the ROC curve, k: Cohen’s kappa index.

**Table 2:** Comparison between in-house ELISA results for *E.coli*-made protein and HEK-made antigen.

		IgG-*E.coli*		
		Positive	Negative	Sum
IgG-HEK	Positive	65	2	67
	Negative	2	78	80
	Sum	67	80	147
	*k* (95% CI) = 0.945 (0.892–0.998)			
		IgM-*E.coli*		
		Positive	Negative	Sum
IgM-HEK	Positive	43	13	56
	Negative	3	25	28
	Sum	46	38	84
	*k* (95% CI) = 0.607 (0.439–0.774)			
